# Roles of plasmid-borne *nod* genes and chromosomal genes of *Bradyrhizobium guangxiense* CCBAU 53363^T^ in peanut nodulation

**DOI:** 10.3389/fpls.2026.1888331

**Published:** 2026-07-20

**Authors:** Jishun Yang, Yue Wu, Dunwei Ci, Zhengfeng Wu, Yongmei Zheng, Tianyi Yu, Juxiang Wu, Shangxia Li, Qiqi Sun, Zhen Yang

**Affiliations:** 1 Shandong Peanut Research Institute, Qingdao, Shandong, China; 2 Shandong Key Laboratory of Peanut Breeding, Qingdao, Shandong, China

**Keywords:** bradyrhizobium, nod genes, peanut, super-nodulation, Tn5 transposon

## Abstract

**Introduction:**

In the symbiotic interaction between legumes and rhizobia, the induction of peanut super-nodulation by Type II *Bradyrhizobium* strains represents a relatively unusual phenomenon, yet the underlying regulatory mechanisms remain largely unclear.

**Methods:**

This study investigated the roles of both *nod* genes and chromosomal genes of the Type II strain *B. guangxiense* CCBAU 53363^T^ (II-53363) in regulating peanut nodulation.

**Results:**

First, all Type II strains harbor identical plasmid-borne *nod* genes, which are distinct from their Type I homologs. Knockout and complementation of *nodB* and *nodC* genes resulted in the loss and restoration, respectively, of the nodulation phenotype in II-53363. Second, through Tn*5* transposon insertion along with targeted gene knockout and complementation, we identified seven chromosomal genes that positively regulate peanut super-nodulation. Disruption of these genes affected the metabolic capacity and EPS production of strain II-53363. Nevertheless, given their absence in some Type II strains and their high sequence similarity to Type I homologs, these chromosomal genes are likely to play an indirect role.

**Discussion:**

This study demonstrates that the plasmid-borne *nod* genes in Type II bradyrhizobia strains are essential for peanut nodulation, although their specific contribution to super-nodulation remains to be demonstrated. Furthermore, it reveals the indirect roles of multiple chromosomal genes in regulating peanut super-nodulation.

## 1 Introduction

Under nitrogen-limited conditions, legume plants interact with rhizobia to form symbiotic nodules, within which rhizobia differentiate into bacteroids and reduce atmospheric nitrogen (N_2_) into ammonium ions (NH_4_^+^) that can be assimilated by legumes. In exchange, legumes supply nutrients (other than nitrogen), energy, and ecological niches required for rhizobial proliferation and nitrogen fixation (Shang et al., 2022). Because the formation of nodules and nitrogen fixation during symbiosis are energetically costly, the number of nodules and their nitrogen-fixing capacity are tightly regulated (Hoffman et al., 2014).

From rhizobial perspective, numerous studies have identified *nod* genes as key determinants of nodulation (Safronova et al., 2023). Initially, flavonoid molecules secreted by legumes activate the rhizobial NodD protein, which in turn triggers the synthesis of Nod factors (NFs). The NF backbone is synthesized by *nodA*, *nodB*, and *nodC* genes, while other genes (e.g., *nodH*, *nodS*, *nodU*, *nodI*, *nodJ*) act as regulatory factors that modify the NF backbone through specific modifications (Liu et al., 2018b). The resulting NFs are then recognized by plant NF receptors, initiating a signaling cascade that ultimately leads to nodule formation. From plant perspective, multiple regulatory systems for nodulation have come into play. First, plant recognition of NFs activates the symbiotic signaling pathway, which in turn promotes rhizobial infection and nodule formation. Within this pathway, receptor-like kinases (DMI2/SYMRK), nucleoporins (NUP133 and NUP85), and nuclear membrane-localized cation channels all contribute to the NF-induced calcium spiking phenomenon (Kosuta et al., 2008). Second, the autoregulation of nodulation (AON) system functions as a feedback mechanism that controls the temporal and spatial susceptibility of the root system to infection, whereby earlier nodulation events inhibit nodulation in younger root tissues (Kassaw et al., 2015). Third, phytohormone signaling pathways have emerged as another important regulator of nodulation. Among these, ethylene negatively regulates infection thread formation and nodule development (Liu et al., 2018a).

Peanut (*Arachis hypogaea* L.) is one of the world’s most important cultivated grain legumes (Chen et al., 2014). The establishment of symbiosis between peanut and *Bradyrhizobium* species occurs via a crack infection pattern at the junctions of hair cells with epidermal and cortical cells (Fabra et al., 2010). The bradyrhizobia involved include *B. arachidis*, *B. guangdongense*, *B. guangxiense*, *B. zhanjiangense*, *B. yuanmingense*, *B. liaoningense*, among others (Chang et al., 2011; Wang et al., 2013; Li et al., 2015; 2019). These peanut-nodulating bradyrhizobia, isolated from the major peanut-producing areas in China (Liaoning province, Liangguang area, and Huanghuaihai area), can be classified into two groups based on symbiotic phenotypes: Type I and Type II. Unlike Type I strains, Type II strains form massive nodules with peanut, with nodule numbers significantly greater—1.5 to 2 times higher—than those of Type I strains. Genomic comparisons reveal that Type I strains possess two copies of *nodD* (*nodD*_1_ and *nodD*_2_), whereas Type II strains have only one (*nodD*_1_). Additionally, the amino acid sequence encoded by *nolA* in Type I strains is 45% longer than that in Type II strains (Li, 2019). However, further study has demonstrated that *nolA* and *nodD*_1_ in Type II strains are not responsible for peanut super-nodulation (Shang et al., 2023). These findings prompt a search for other factors contributing to this super-nodulation phenomenon.

Given the absence of systematic nucleotide sequence functional analysis of Type II bradyrhizobia, we hypothesize that certain genes may serve as key factors underlying this super-nodulation phenotype. In this study, *B. zhanjiangense* CCBAU 51778 (designated as I-51778, representing Type I bradyrhizobia) and *B. guangxiense* CCBAU 53363 (designated as II-53363, representing Type II bradyrhizobia) were selected as model strains. The primary objective of this study was to investigate the genetic basis of how Type II bradyrhizobia regulate peanut nodulation from the rhizobial perspective. By doing so, we provide a fundamental framework for elucidating the mechanisms underlying super-nodulation in the peanut-rhizobia symbiosis.

## 2 Materials and methods

### 2.1 Bacteria and culture condition

The bacterial strains and plasmids used in this study are listed in **Supplementary Table 1** (Figurski and Helinski, 1979; Quandt and Hynes, 1993). A total of 647 Tn*5* transposon-inserted mutants of II-53363 were obtained from a previously constructed Tn*5* mutant library (Wu et al., 2020). *Escherichia coli* and rhizobia were cultured in Luria-Bertani (LB) medium at 37 °C (Sambrook et al., 1989) and tryptone yeast (TY) medium at 28 °C (Beringer, 1974), respectively.

### 2.2 The statistical analysis of peanut symbiotic phenotypes with II-53363 and I-51778

Peanut seeds were surface-sterilized by immersion in 75% (v/v) ethanol for 1 min, followed by immersion in 5% (w/v) NaClO for 10 min. The seeds were then rinsed eight times with sterile distilled water, transferred onto 0.6% agar-water plates, and germinated for 5 days at 25 °C in darkness. The resulting seedlings were planted in Leonard jar assemblies containing sterile vermiculite moistened with low-nitrogen plant nutrient solution (Vincent, 1970). Each seedling was inoculated either with 0.8% NaCl solution (uninoculated control) or with 1 mL of rhizobial suspension adjusted to an OD_600_ of 0.2. The plants were grown in a greenhouse at 25 °C with a 12 h light/12 h dark photoperiod (LED lighting). To assess the symbiotic phenotypes of peanut inoculated with II-53363 and I-51778, peanut chlorophyll content, shoot dry weight, nodule number, and nodule fresh weight were measured at 13, 23, 33, 43, and 53 days post-inoculation (dpi) (Wu et al., 2020). 3 plants per treatment were sampled each time.

### 2.3 II-53633 knockout and complementation of the *nod* genes and their association with peanut phenotype

The PCR primers used for plasmid construction, gene knockout, and gene complementation are listed in **Supplementary Table 1**. Methods for DNA fragment amplification, pJQ200SK plasmid construction, and gene knockout were as described in Shang et al. (2023). Taking the knockout of *nodB* gene as an example, the specific steps were as follows: (1) Construction of the gene knockout vector: the upstream and downstream fragments of II-53363 *nodB* were amplified and ligated into the linearized pJQ200SK vector using the Seamless Cloning Kit (Tiangen Biochemical Technology Co., Ltd., Beijing, China) according to the manufacturer’s protocol. The ligation products were transformed into *E. coli* competent cells, and successful recombinants were identified by PCR verification of the inserted fragments. (2) Knockout of *nodB* gene: the *nodB* gene of II-53363 was knocked out via triparental conjugation, with II-53363 as the recipient strain, the recombinant pJQ200SK plasmid (containing the upstream and downstream fragments of II-53363 *nodB*) as the donor strain, and pRK2013 as the helper strain. After homologous recombination and three rounds of purification screening, the *nodB* knockout mutant, designated as 53ΔnodB, was obtained. According to this method, we obtained both gene knockout (53ΔnodB, 53ΔnodC) and gene complementation mutants (53ΔnodB+nodB, 53ΔnodC+nodC).

To record the effects of II-53363 *nodB* and *nodC* genes on peanut symbiotic phenotypes, strains of II-53363, 53ΔnodB, 53ΔnodC, 53ΔnodB+nodB, and 53ΔnodC+nodC were inoculated with sterile peanuts. Peanut chlorophyll content, shoot dry weight, nodule number, and nodule fresh weight were recorded at 13, 23, 33, and 43 dpi. 3 plants per treatment were sampled each time.

### 2.4 Screening of Tn*5* transposon insertion mutants and corresponding genes knockout

The 647 Tn*5* transposon inserted mutants of II-53363 were derived from the study of Wu et al. (2020). For the selection of Tn*5* insertion mutants, sterile peanuts were inoculated with II-53363 and each of the 647 Tn*5* insertion mutants. Nodule numbers were recorded at 43 dpi. Each treatment consisted of 9 plants. 15 Tn*5* insertion mutants that formed significantly fewer nodules than II-53363 were selected.

To eliminate the polar effects caused by Tn*5* transposon insertion, it is necessary to completely knock out the genes containing the Tn*5* insertion and then complement the knocked-out genes, followed by examining the symbiotic phenotypes of these mutants with peanut. First, to determine the insertion sites of Tn*5* transposon and to generate gene knockout mutants, we amplified and sequenced the insertion regions (the target gene) using PM PCR primer (**Supplementary Table 1**) according to the method of Liu et al. (2018b). Second, the sequence of the target gene and its upstream and downstream fragments were retrieved from the National Center for Biotechnology Information (NCBI) database (https://blast.ncbi.nlm.nih.gov/Blast.cgi) following the approach of Wu et al. (2020). Third, the target gene was knocked out and complemented via triparental conjugation. For gene knock out, II-53363 was used as the recipient strain, the recombinant pJQ200SK plasmid (containing the upstream and downstream fragments of the target gene) served as the donor, and pRK2013 was used as the helper strain. For gene complementation, the gene knock out mutant was used as the recipient strain, the recombinant pJQ200SK plasmid (carrying the target gene along with its upstream and downstream fragments) served as the donor, and pRK2013 was used as the helper strain. Following homologous recombination and three rounds of purification, 15 gene knockout mutants and their corresponding 15 gene-complemented mutants were successfully obtained. Last, sterile peanuts were inoculated with the wild-type strain II-53363, the 15 selected Tn*5* insertion mutants, and their corresponding 15 gene knockout and gene-complemented mutants. Nodule numbers were recorded at 43 dpi. Each treatment consisted of 9 plants. Based on these results, seven pairs of mutants that exhibited significantly reduced nodule numbers without polarity effects were selected. Seven pairs of mutants included Tn*5* transposon inserted mutants (H10-T, H33-T, H41-T, L82-T, H172-T, L265-T, L646-T), gene knockout mutants (H10-P, H33-P, H41-P, L82-P, H172-P, L265-P, L646-P), and gene-complemented mutants (H10-P+H10, H33-P+H33, H41-P+H41, L82-P+L82, H172-P+H172, L265-P+L265, L646-P+L646).

### 2.5 Rhizobial free-living characteristics

Biolog Gen III microplates (containing 71 carbon sources and 23 chemosensitive reagents; Biolog, Hayward, CA, USA) were used according to the manufacturer’s instructions. Rhizobia were collected and adjusted to an optical density (OD_600_) of 0.12. Then, 80 μl the rhizobial suspension was inoculated into each well and cultured at 28 °C for 15 days. Color changes in each well were detected at OD_750_ (Janczarek and Rachwał, 2013). Each treatment was performed in triplicate.

To determine the rhizobial generation time, growth curves were established. Briefly, strains I-51778, II-53363, and the gene knockout mutants were cultured in TY liquid medium to the logarithmic phase (OD_600_ = 0.6) and then adjusted to OD_600_ = 0.03 using sterile TY liquid medium. The adjusted suspensions were cultured at 28 °C in a 100-well test plate (400 μL per well) using a microbial growth curve meter (Bioscreen C, Oy Growth Ab Ltd). Cell densities were measured at OD_600_ every 6 hours (h) over a total period of 96 h. The operational procedures and growth curve plotting were performed according to the Bioscreen C manufacturer’s instructions. The generation time was calculated from the growth curve using the formula: Generation time = Log2(t_2_ - t_1_)/(LogW_2_ - LogW_1_), where W_1_ and W_2_ represent cell concentrations at 30 h (t_1_) and 36 h (t_2_), respectively. Each treatment consisted of 9 samples.

EPS yield was measured using the anthrone-H_2_SO_4_ method (Tomlinson et al., 2010). In brief, 2 ml of rhizobial suspension (OD_600_ = 0.6) was inoculated into 100 ml TY liquid medium. The strains were cultured to the stationary phase (OD_600_ = 1.2), after which the supernatants were collected for measurement. Each treatment consisted of 9 samples.

### 2.6 Phylogenetic analysis of *nod* and Tn*5* insertion genes

Type I bradyrhizobia used in this part included *Bradyrhizobium* sp. CCBAU 21365 (CP030036), *Bradyrhizobium* sp. CCBAU 51753 (CP030037), *Bradyrhizobium* sp. CCBAU 51765 (CP030038), *B. zhanjiangense* CCBAU 51778 (CP022221), and *Bradyrhizobium* sp. CCBAU 53421 (CP030047). Type II bradyrhizobia used in this part included *B. guangdongense* CCBAU 51649 (CP030052), *B. guangdongense* CCBAU 51658 (CP030058), *B. guangzhouense* CCBAU 51670 (CP030054), *Bradyrhizobium* sp. CCBAU 53338 (CP030049), *Bradyrhizobium* sp. CCBAU 53340 (CP030056), *Bradyrhizobium* sp. CCBAU 53351 (CP030060), and *B. guangxiense* CCBAU 53363 (CP022220). All genome sequence data are available in the NCBI database under the indicated accession numbers. The nucleotide sequences of *nod* genes, Tn*5* transposon-inserted genes, 16S rDNA, and housekeeping genes (*atpD* and *recA*) from II-53363 were used as references to identify homologous sequences in the Type I and Type II strains. Sequence identity percentages were calculated using the Poisson correction model in MEGA 5.0 (Wu et al., 2020). Whole-genome alignments between I-51778 and II-53363, as well as alignments of symbiotic plasmids from Type II bradyrhizobia, were performed using MAUVE software.

### 2.7 Statistical analysis

Data presented in bar charts were shown as mean ± standard deviation (SD). Statistical analyses were performed using SPSS software (version 20.0; SPSS Research Institute, Inc., USA). Significant differences among treatments were determined by one-way analysis of variance (ANOVA) followed by Duncan’s multiple range test. For the analysis of symbiotic phenotypes in peanuts inoculated with I-51778, II-53363, 53Δ*nodB*, 53Δ*nodC*, 53Δ*nodB*+*nodB*, and 53Δ*nodC*+*nodC* (n = 3), significance levels are indicated as *p < 0.05, **p < 0.01, ***p < 0.001, ****p < 0.0001. For the analysis of nodule number in peanuts inoculated with II-53363, H10-T/P, H33-T/P, H41-T/P, L82-T/P, H172-T/P, L265-T/P, L646-T/P, H10-P+H10, H33-P+H33, H41-P+H41, L82-P+L82, H172-P+H172, L265-P+L265, L646-P+L646 (n = 9), a *p*-value < 0.05 was considered statistically significant. For the analysis of rhizobial generation time and EPS yield across different treatments (n = 9), a *p*-value < 0.05 was considered statistically significant.

## 3 Results

### 3.1 Symbiotic properties of II-53363 and I-51778 associated peanut

**Figure 1:**
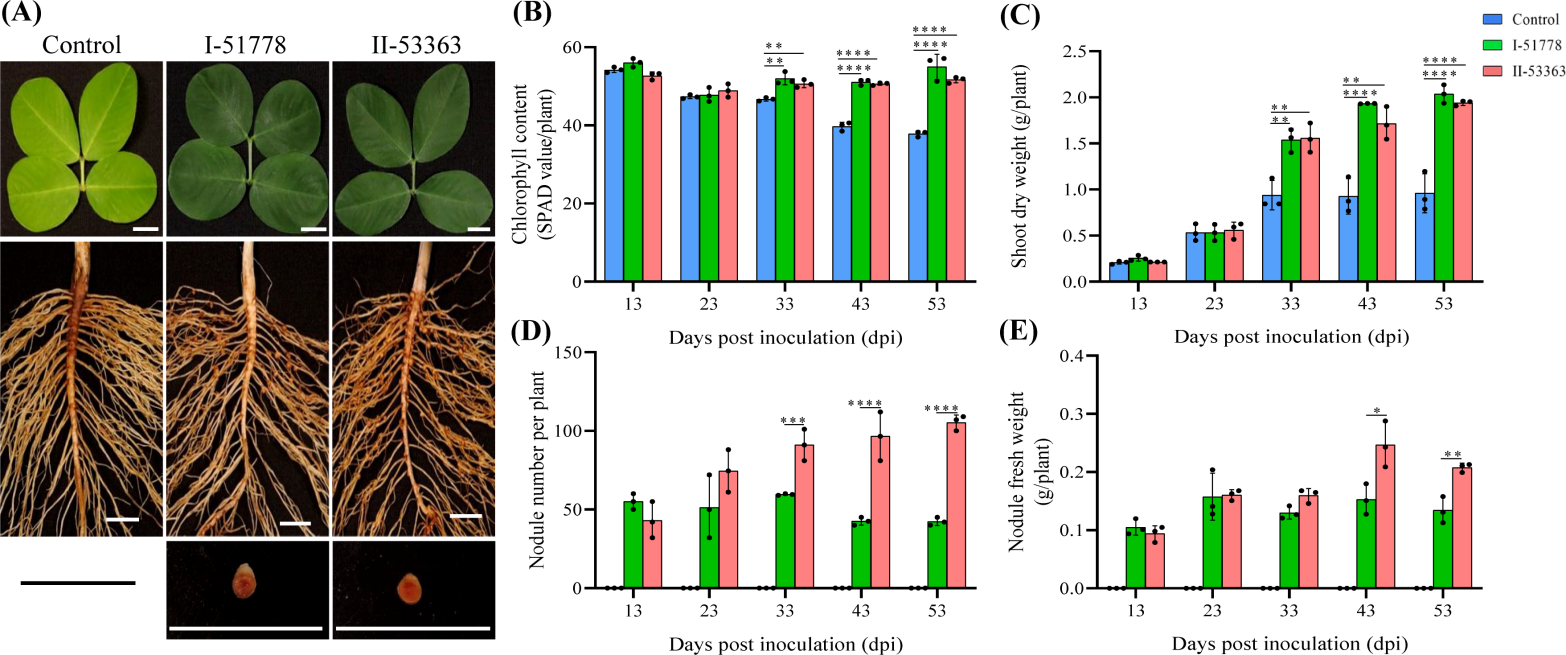
Symbiotic phenotypes of peanut inoculated with I-51778 and II-53363 at different days post inoculation (dpi). **(A)** Symbiotic phenotypes. **(B)** Chlorophyll content. **(C)** Shoot dry weight. **(D)** Nodule number per plant. **(E)** Nodule fresh weight. Scale bar = 1 cm. Data are mean ± SD (Duncan’s multiple range test, n = 3, *p < 0.05, **p < 0.01, ***p < 0.001, ****p < 0.0001).

Peanut symbiotic phenotypes were recorded from 13 to 53 dpi following inoculation with I-51778 and II-53363 (**Figure 1**). After 33 dpi, compared with the control group, peanut chlorophyll content and shoot dry weight remained higher in I-51778 and II-53363 treatments. However, no significant differences in chlorophyll content or stem dry weight were observed between the two treatments (**Figures 1A, B**). Notably, the number and fresh weight of nodules in II-53363 were significantly higher than those in I-51778 after 33 dpi and 43 dpi, with increases ranging from 52.94% to 148.82% and from 54.98% to 61.43%, respectively (**Figures 1C, D**). Overall, compared with I-51778, the most distinctive symbiotic phenotype of II-53363 inoculation is the formation of an extremely large number of nodules.

### 3.2 *nod* genes of II-53363 influence peanut nodulation

**Figure 2:**
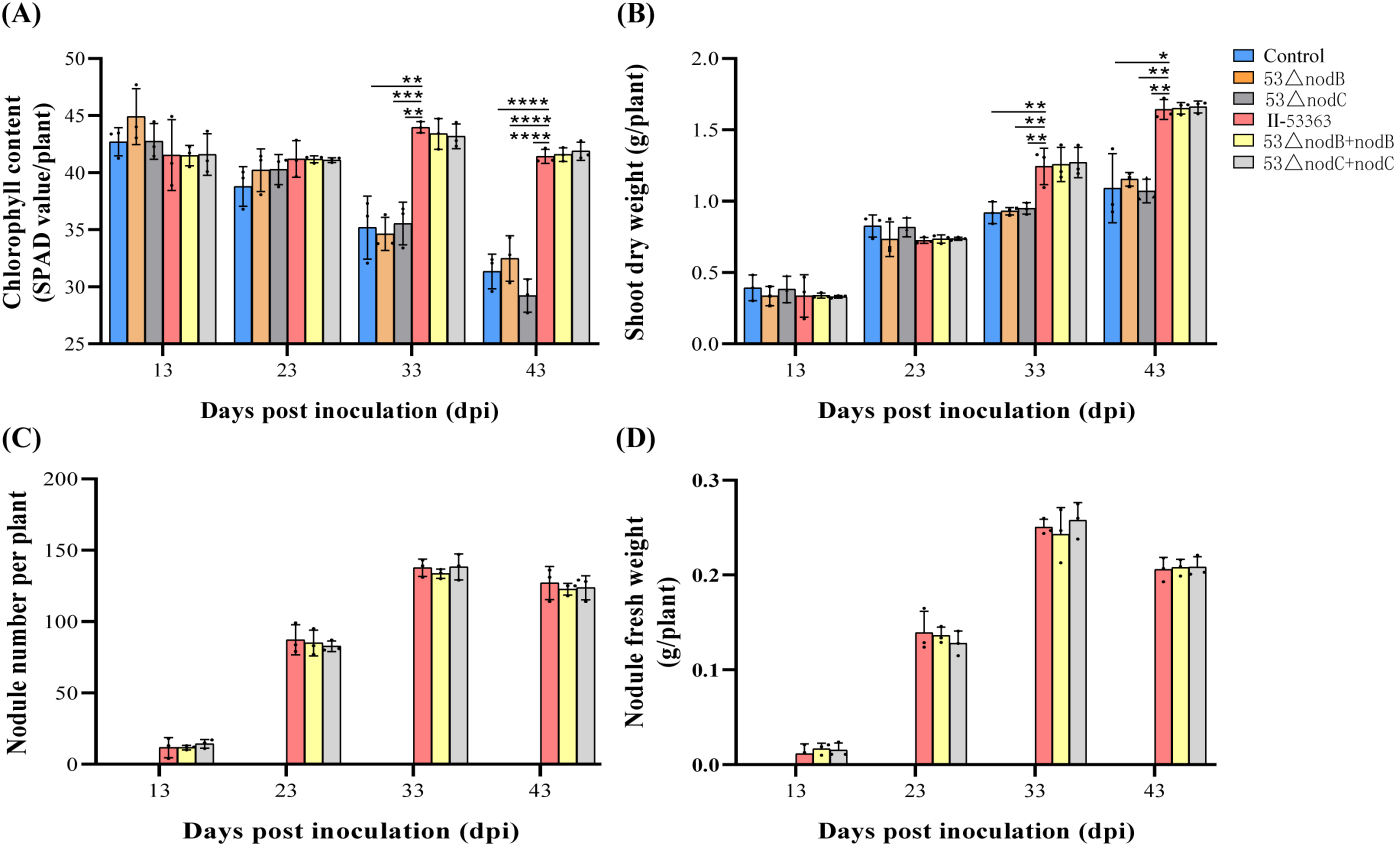
Symbiotic phenotypes of peanut inoculated with II-53363, 53ΔnodB, 53ΔnodC, 53ΔnodB+nodB, and 53ΔnodC+nodC at different dpi. **(A)** Chlorophyll content. **(B)** Shoot dry weight. **(C)**:Nodule number per plant. **(D)** Nodule fresh weight. Data are mean ± SD (Duncan’s multiple range test, n = 3, *p < 0.05, **p < 0.01, **p < 0.001, ****p < 0.0001).

To investigate the contribution of II-53363 *nod* genes to peanut super-nodulation, we selected the *nodB* and *nodC* genes as representative examples. The resulting knockout mutants, designated 53ΔnodB and 53ΔnodC, failed to elicit nodule formation on peanut plants. In addition, relative to the wild-type II-53363, inoculation with either mutant led to decreased chlorophyll content and shoot dry weight after 33 dpi (**Figure 2**). Collectively, these results demonstrate that the *nod* genes of II-53363 are indispensable for peanut effective nodulation. Subsequently, the *nodB* and *nodC* genes of II-53363 were reintroduced into the 53ΔnodB and 53ΔnodC mutants, generating the complementation strains 53ΔnodB+nodB and 53ΔnodC+nodC, respectively. And the symbiotic phenotypes—including chlorophyll content, shoot dry weight, nodule number, and nodule fresh weight—induced by 53ΔnodB+nodB and 53ΔnodC+nodC mutant strains in peanut were indistinguishable from those induced by II-53363 (**Figure 2**). Taken together, these results indicate that the *nod* genes of II-53363 are essential for peanut nodulation.

### 3.3 Other super-nodulation-regulating genes of II-53363

**Figure 3:**
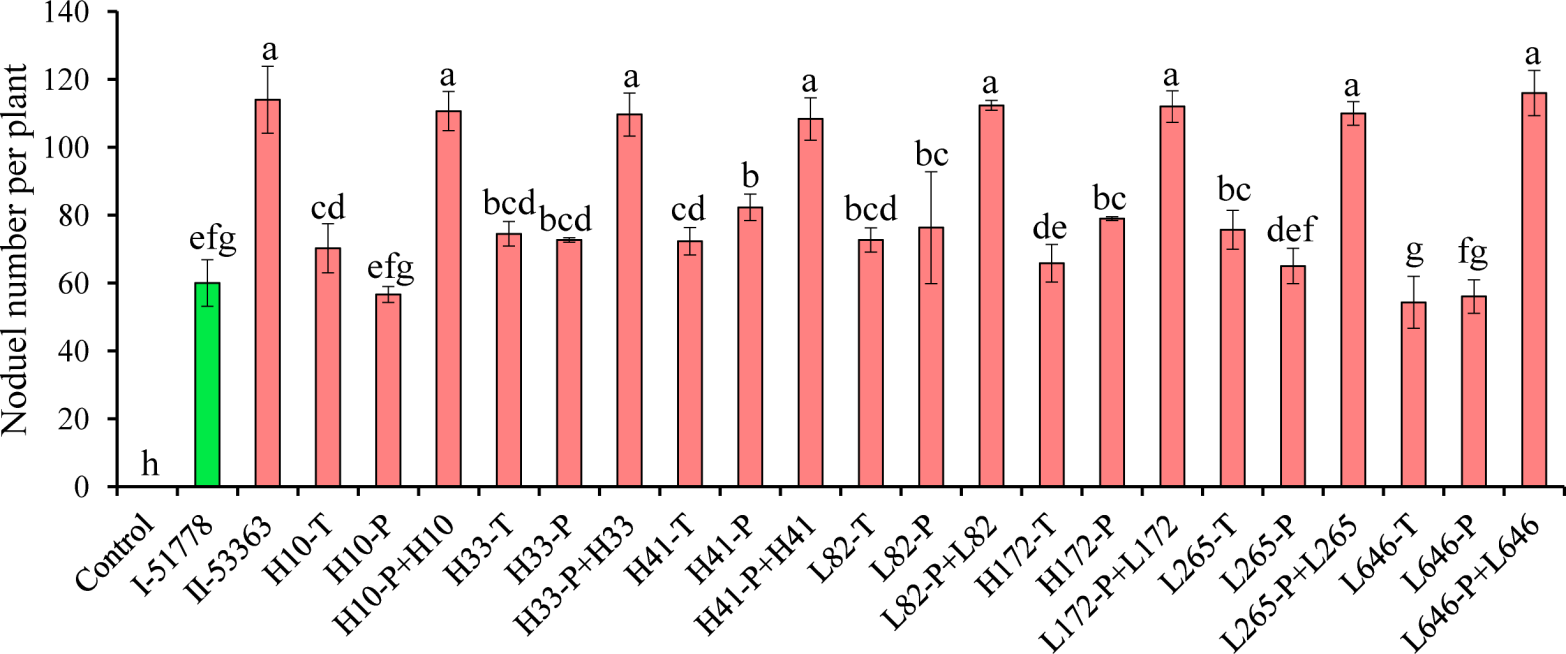
Peanut nodule numbers at 43 dpi following inoculation with I-51778, II-53363, II-53363-derived Tn*5* insertion mutants (gene number-T), corresponding gene knockout mutants (gene number-P), and their complemented derivatives (gene number-P+gene number). Data are mean ± SD, different letters indicated significant difference (Duncan’s multiple range test, n = 9, *p* < 0.05).

To investigate the roles of chromosomal genes in regulating peanut nodulation, we screened the Tn*5* transposon insertion mutant library of II-53363 for mutants with altered nodule numbers. Among 647 insertion mutants (Wu et al., 2020), 15 mutants with significantly lower nodule numbers than II-53363 were identified. These candidate genes were then subjected to independent knockout to rule out any polarity effects on nodulation. For seven of these genes—*H10*, *H33*, *H41*, *L82*, *H172*, *L265*, and *L646*—both Tn*5* insertion (mutant-T) and targeted gene knockout (mutant-P) mutants led to a marked reduction in nodule number compared to II-53363. Meanwhile, after inoculation with the gene-complemented mutants (H10+H10, H33-P+H33, H41-P+H41, L82-P+L82, H172-P+H172, L265-P+L265, L646-P+L646), peanut plants restored the super-nodulation trait (**Figure 3**). These results demonstrate that these seven genes from II-53363 act as positive regulators of the super-nodulation phenotype in peanut.

**Figure 4:**
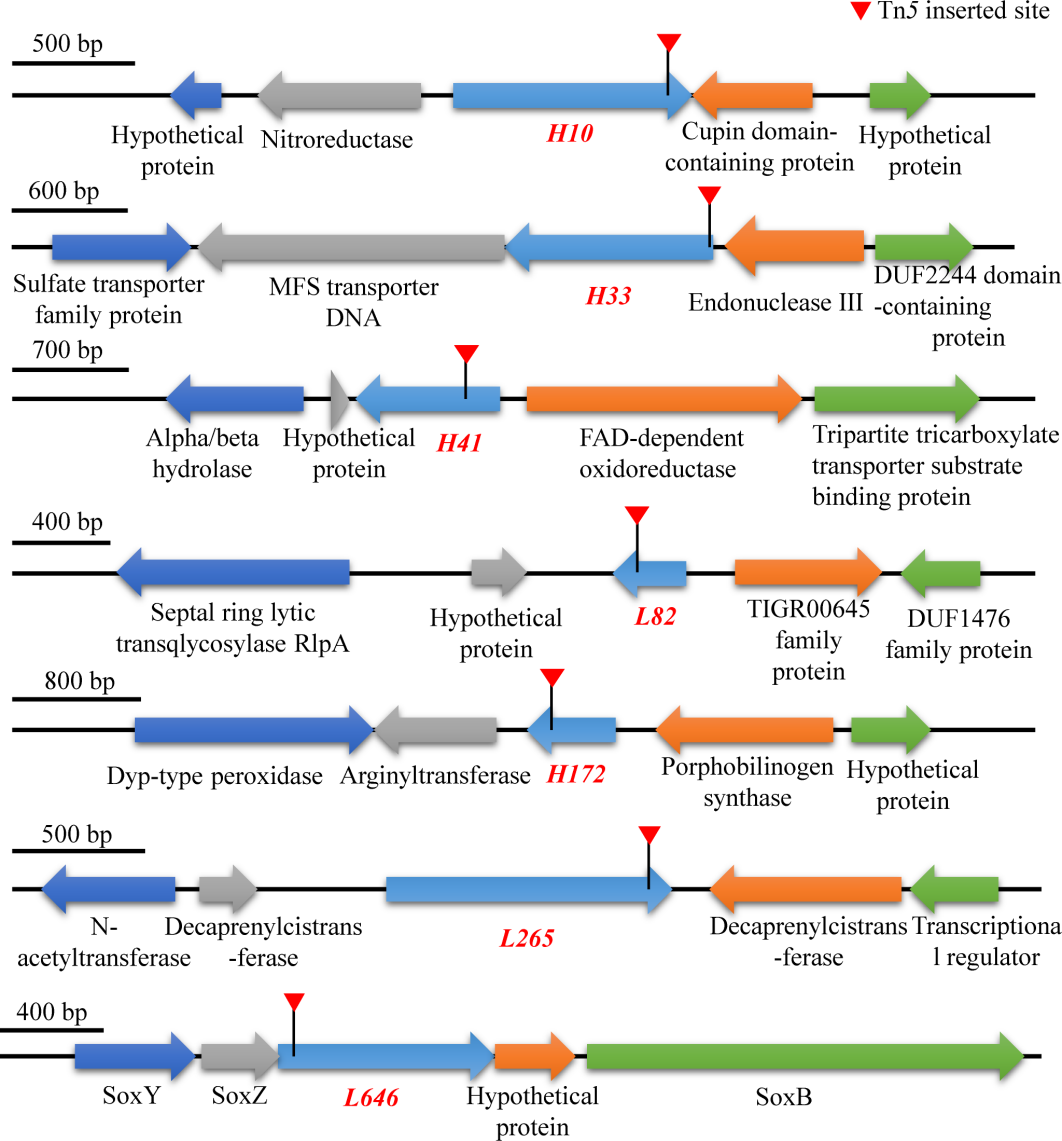
Physical map of Tn*5* inserted genes (inverted triangle) in II-53363 genome. Each inserted gene is flanked by two adjacent genes. The direction of the arrows indicates the transcriptional orientation of the genes. Functions of these genes are displayed below the arrows.

To detect the functions of these mutated genes, we analyzed their characteristics, including length, genomic location, protein annotation and accession, sequence identity to known proteins, and neighboring genes (**Figure 4**; **Supplementary Table 2**). All mutated genes reside on II-53363 chromosome. *H10* (962 bp) encodes an alpha/beta hydrolase family lipase/esterase, sharing 94.2% identity with proteins from *Bradyrhizobium* sp. TSA1 and DOA1. *H33* (1134 bp) encodes an HlyD family secretion protein with 94.5% identity to that of *Bradyrhizobium* sp. BK707. *H41* (911 bp) encodes LysR family transcriptional regulator (94.1% identity to *B. huanghuaihaiense* CGMCC 1.10948). *L82* (291 bp) encodes a hypothetical protein (86.6% identity to *Bradyrhizobium* sp. CNPSo 3426). *H172* (542 bp) encodes an RDD family protein (97.2% identity to *B. huanghuaihaiense* CGMCC 1.10948). *L265* (1113 bp) encodes a putative acyltransferase, with only 35.1% identity to a known acyltransferase from *Bradyrhizobium* sp. UNPF46. *L646* (867 bp) encodes sulfur oxidation c-type cytochrome SoxA (95.4% identity to *Bradyrhizobium* geno sp. SA-4).

### 3.4 Free-living characteristics of mutants

**Figure 5:**
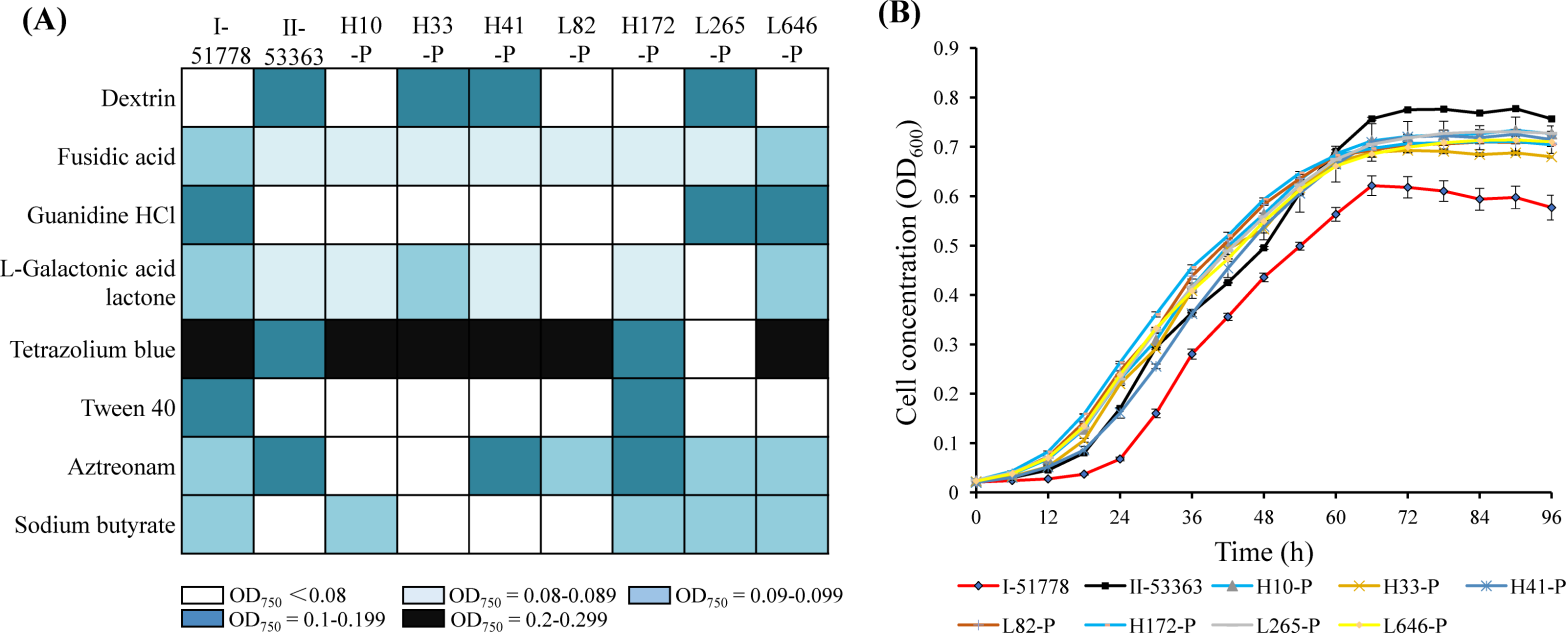
Metabolic capacity **(A)** and growth curves **(B)** of I-51778, II-53363, and II-53363-derived gene knockout mutants (designated as gene number-P) under free-living conditions. In **(A)**, the relative growth status of each strain was assessed using three microplates and is represented by color intensity.

To determine whether the free-living characteristics of these mutants had been altered and whether such changes were related to the decreased nodule numbers, the metabolic capacity, growth curves, and EPS yields of I-51778, II-53363, and seven gene knockout mutants were examined. Results from the Biolog test kit showed that the metabolic capacity of these mutants varied to different degrees (**Figure 5A**). Among them, several metabolic substrates differed between the mutants and II-53363 but were similar to those of I-51778, including Dextrin (H10-P, L82-P, H172-P, L646-P), Fusidic acid (L646-P), Guanidine HCl (L265-P, L646-P), L-Galactonic acid lactone (H33-P, L646-P), Tetrazolium blue (H10-P, H33-P, H41-P, L82-P, L646-P), Tween 40 (H172-P), Aztreonam (L82-P, L265-P, L646-P), and Sodium butyrate (H10-P, H172-P, L265-P, L646-P). Overall, the assimilation of Dextrin, Tetrazolium blue, Aztreonam, and Sodium butyrate was affected in most mutants, suggesting that these altered metabolic abilities may play a positive role in reducing peanut nodule numbers.

The generation times of the strains were calculated based on their growth curves (**Figure 5B**; **Supplementary Table 3**). Among them, the generation time of II-53363 (19.4 h) was significantly longer than that of I-51778 (6.8 h). The generation times of H10-P, H33-P, H41-P, and L82-P mutants (12.2 – 14.5 h) were considerably shorter than that of II-53363, indicating a negative regulatory relationship between the mutated genes and the growth rate of II-53363. The generation times of H172-P, L265-P, and L646-P (18.1–20.3 h) were similar to that of II-53363, suggesting that these three genes had no effect on the growth rate of II-53363. Based on these findings, some genes were found to affect the growth rate of II-53363, while others did not. However, since all mutants reduced the number of peanut nodules, it can be inferred that the growth rate of II-53363 is unlikely to be closely related to peanut super-nodulation.

The EPS yield of II-53363 was significantly lower than that of I-51778, whereas the EPS yields of these mutants increased to the level of I-51778 or above (**Supplementary Table 3**). Therefore, a negative correlation may exist between the EPS yield of peanut bradyrhizobia and peanut nodule number. The lower EPS yield of II-53363 may contribute to the super-nodulation observed in peanuts inoculated with II-53363.

### 3.5 Characteristics of nodulation regulatory genes of II-53363

**Figure 6:**
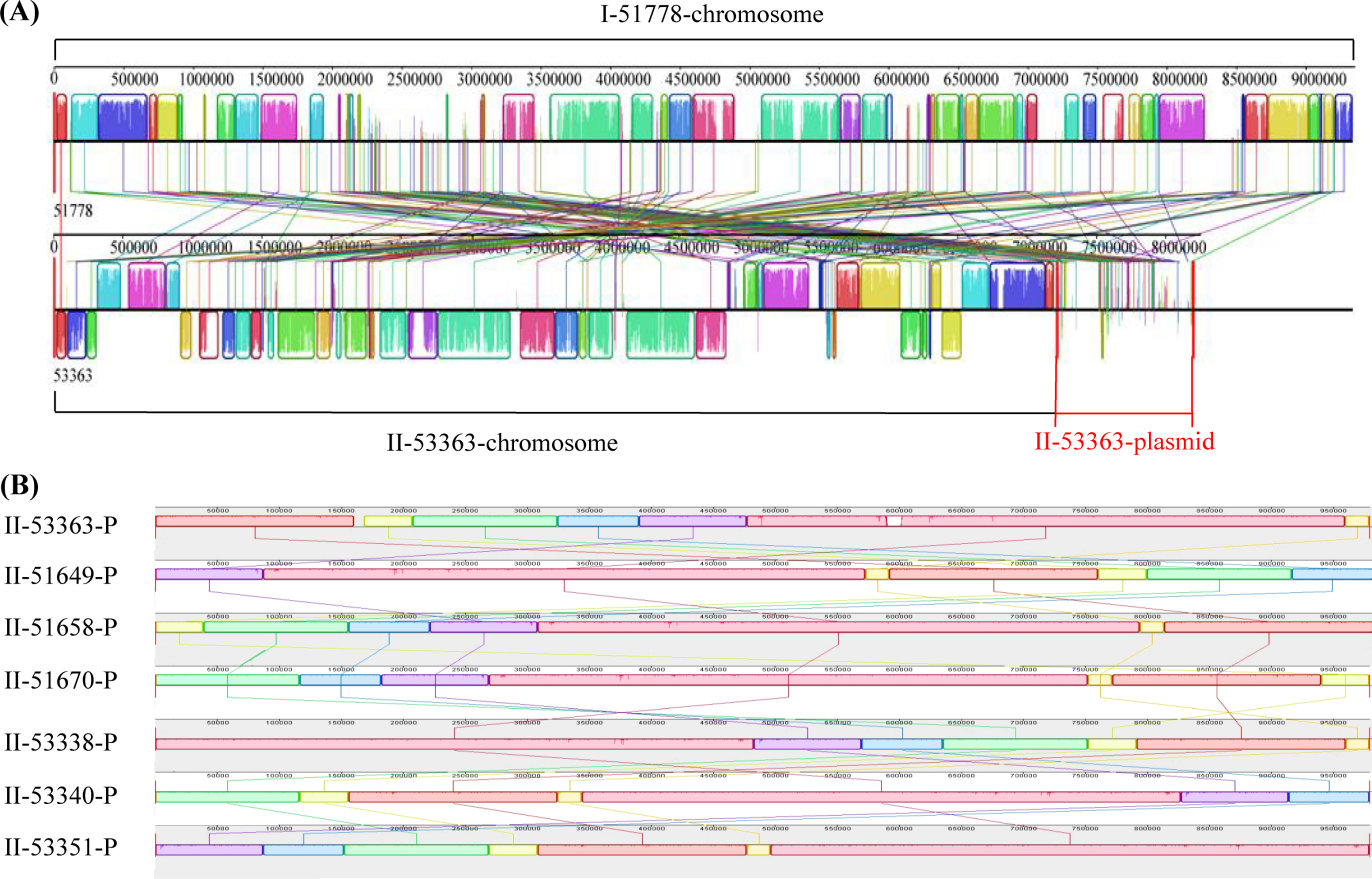
Genomic alignment of strains I-51778 and II-53363 **(A)**, and the symbiotic plasmids of Type II bradyrhizobia **(B)**.

Genome alignment between I-51778 and II-53363 showed that both strains carried a single chromosome, but the symbiotic plasmid was unique to II-53363. While chromosomal genes were highly similar between the two strains, the symbiotic plasmid genes of II-53363 differed markedly from their homologs in I-51778 (**Figure 6A**). Additionally, the Tn*5* transposon-inserted genes, 16S rDNA, and housekeeping genes resided on the chromosome of II-53363 (**Supplementary Table 4**).

Using II-53363 as a reference strain, we compared the above genes with those of other Type I and Type II bradyrhizobia. Nucleotide alignment revealed that all Type II strains shared an identical symbiotic plasmid (**Figure 6B**), with their *nod* gene sequences showing 100% similarity to those of II-53363. In marked contrast, the similarity of *nod* genes between II-53363 and Type I strains was substantially lower, ranging from only 39.4% to 61.2% (**Supplementary Table 4**). The complete concordance between *nod* gene characteristics of Type II strains and their ability to induce peanut super-nodulation suggests that the *nod* genes of II-53363 may be key regulators of this phenotype. Therefore, the future experiments should focus on examining the effects of *nod* genes on peanut super-nodulation.

For chromosomal genes, the 16S rDNA and housekeeping genes of II-53363 exhibited 72.2% – 96.1% similarity to Type I homologs and 82.7% – 96.6% similarity to Type II homologs. Similarly, its Tn*5* transposon-inserted genes showed 68.5% – 92.2% similarity to Type I homologs and 77.1% – 94.4% similarity to Type II homologs. Among these, the *L265* of II-53363 was absent from both Type I and Type II strains. *H172* and *L646* were present on the chromosomes of both strain types, whereas *H10*, *H33*, *H41*, and *L82* were found only in certain strains of Type I and Type II (**Supplementary Table 4**). Overall, the Tn*5* transposon-inserted genes of II-53363 showed high sequence similarity to their homologs in both Type I and Type II strains, yet not all Type I or Type II strains carried these homologous genes. This distribution pattern is inconsistent with the observation that all Type II strains are capable of affecting peanut super-nodulation. Therefore, we infer that these Tn*5* transposon-inserted genes are not the key determinants of the ability of II-53363 to influence peanut super-nodulation.

## 4 Discussion

Nodules represent the most distinctive symbol of symbiosis between rhizobia and legumes. Earlier study has revealed that certain *B. japonicum* strains, including NKS4, NKM2 and NKTG2, exhibit the superior nodulation on soybean (Nguyen et al., 2020). Similarly, in this study, the symbiotic interaction between peanut and Type II bradyrhizobia, exemplified by strain II-53363, also displayed a super-nodulation phenotype. However, at present, the underlying mechanisms driving this super-nodulation in peanut remain elusive. To determine the potential role of Type II strains in modulating peanut super-nodulation, we analyzed the genomic characteristics of Type II strains and investigated the effects of their *nod* genes and Tn*5* transposon-randomly inserted chromosomal genes on peanut super-nodulation.

### 4.1 Effects of type II bradyrhizobial *nod* genes on peanut nodulation

For Type II bradyrhizobial genome, *nod* genes were found on the symbiotic plasmid, and their nucleotide sequences exhibited significant differences from those of homologous genes in Type I bradyrhizobia (**Supplementary Table 4**). Given the crucial roles that *nod* genes regulate legume nodulation, we inferred that these genes were essential for inducing peanut super-nodulation. Further researches revealed that the knockout of *nodB* and *nodC* genes (53ΔnodB and 53ΔnodC) completely hindered the nodulation of peanut, and the gene-complemented mutants (53ΔnodB+nodB and 53ΔnodC+nodC) restored the super-nodulation phenotype of peanut (**Figure 2D**). In summary, this study demonstrates that the *nod* genes carried on the plasmid of Type II bradyrhizobia regulate normal nodulation in peanut plants, but does not provide evidence that these genes specifically govern the super-nodulation. Therefore, future researches should focus on elucidating the role of *nod* genes in regulating peanut super-nodulation. Specifically, efforts may include swapping the *nod* gene cluster between Type I and Type II bradyrhizobia, directly analyzing the structural differences in NFs produced by these two rhizobial types, and examining how such differences affect peanut super-nodulation. Furthermore, this result suggests that NFs produced by Type II bradyrhizobia are critical for peanut nodulation. A previous study demonstrated that knock out of *nodD*1 gene, a gene regulating NF synthesis, does not impair the super-nodulation between II-53363 and peanut (Shang et al., 2023). Based on this finding and our demonstration of the importance of II-53363 NFs in peanut nodulation, we propose that an alternative regulatory pathway for NF synthesis, independent of *nodD1*, operates during the super-nodulation.

### 4.2 Effects of type II bradyrhizobial Tn*5* transposon-inserted genes on peanut super-nodulation

To genome-wide screen for genes in Type II bradyrhizobia that induce peanut super-nodulation, we used Tn*5* transposon random insertion and successfully isolated multiple chromosomal genes involved in regulating this trait. Among these, the chromosomal genes *H10*, *H33*, *H41*, *H172*, and *L646* of II-53363 strain were found to significantly reduce peanut nodule numbers (**Figure 3**). Physiological and biochemical analyses revealed that mutations in these genes affected the metabolic function and EPS production of II-53363 (**Figure 5**). However, given that some of these genes are absent in Type II rhizobia (**Supplementary Table 4**), we infer that these chromosomal genes may be regulated by other key genes and thus indirectly affect the super-nodulation phenotype.

Based on a review of the relevant literature, we inferred the functions of these genes in regulating peanut super-nodulation. *H10* encodes a lipase/esterase involved in lipid transport or metabolism, which promotes bacterial infection of plant cells by either depolymerizing cell wall components or modifying lipid-based signaling molecules—such as jasmonic acid—to attenuate plant defense (Mastronunzio et al., 2008; Liu et al., 2018a). *H33* encodes HlyD, a membrane fusion protein of the type 1 secretion system (T1SS), which functions primarily as a host-specific recognition factor in rhizobia to regulate host range (Scheu et al., 1992; Yan et al., 2017). H41 protein belongs to the LysR-type transcriptional regulator (LTTR) family and functions as a DNA-binding protein in the interaction between rhizobia and leguminous plants, similar to NodD and SyrM (symbiotic regulators) (Rostas et al., 1986; Schell, 1993; Acosta-Jurado et al., 2020). Both proteins are responsible for regulating the expression of *nod* genes to form NFs. *H172* encodes an RDD (arginine-aspartate-aspartate) family protein, which functions as a novel Na^+^(Li^+^, K^+^)/H^+^ antiporter that confers bacterial tolerance to alkaline and osmotic fluctuations in plant cells (Slonczewski et al., 2009; Shao et al., 2018). *L646* encodes SoxA, a sulfur-oxidizing c-type cytochrome that primarily provides sulfur for the synthesis of NFs in rhizobia (Tate et al., 1997; Kilmartin et al., 2016).

The above discussion suggests that II-53363 may employs multiple mechanisms to influence peanut nodule number: regulating rhizobial colonization on peanut roots via T1SS (*H33*), affecting NFs synthesis through LTTR (*H41*) and sulfur metabolism system (*L646*), facilitating rhizobial infection by secreting lipase/esterase (*H10*); and enhancing rhizobial resistance to plant defense responses via the Na^+^(Li^+^, K^+^)/H^+^ antiporter (*H172*). In addition, from a physiological and biochemical perspective, we found that these genes also affect the metabolic function and EPS production of free-living II-53363, which may in turn play a role in regulating peanut nodule number. Further studies should focus on whether these physiological and biochemical changes influence the attachment of rhizobia to the peanut rhizosphere, thereby affecting peanut nodulation. However, when we attempted to label the strains with GFP or RFP, the fluorescent proteins were not stably inherited and were completely lost after several subcultures. Therefore, achieving stable fluorescent labeling of peanut rhizobia and monitoring the root colonization patterns of mutant strains represents a key challenge to be addressed in our subsequent experiments. On the other hand, to date, only two rhizobial factors have been reported to regulate nodule number: gibberellins synthesized by *Mesorhizobium loti* in *Lotus japonicus*, and tRNA-derived small RNAs of *Bradyrhizobium japonicum* USDA 110 in *Glycine max* (Ren et al., 2019; Tatsukami and Ueda, 2016). Therefore, although further studies are required to validate our findings, this study provides the first indication of a link between rhizobial factors and host nodulation regulatory systems.

## 5 Conclusion

This study revealed that Type II bradyrhizobial strain CCBAU 53363^T^ is capable of inducing super-nodulation in peanut, the plasmid-borne *nodB* and *nodC* genes are critical for peanut nodulation, and that seven chromosomal genes positively correlate with peanut super-nodulation. These results provide a basis for proposing a model of rhizobial regulation of nodule number in leguminous plants. In subsequent studies, we will examine the regulatory roles of *nod* genes in peanut super-nodulation and the correlation between *nod* genes and chromosomal mutation genes. Additionally, by fluorescently labeling knockout mutants of the chromosomal genes, we will dissect the mechanisms underlying the reduced nodule numbers caused by these mutations.

## Data Availability

The datasets presented in this study can be found in online repositories. The names of the repository/repositories and accession number(s) can be found in the article/**Supplementary Material**.

## Supplementary material

Supplementary Table 1 Bacteria, plasmids and primers used in this study.

Supplementary Table 2 Characteristics of seven mutated genes of II-53363.

Supplementary Table 3 Generation time and EPS yield of I-51778, II-53363 and gene knockout mutants of II-53363 (gene number-P) in free-living conditions.

Supplementary Table 4 II-53363 *nod* genes, Tn*5* transposon-inserted genes and their similarity percentages with homologous genes of Type I and Type II bradyrhizobia.

## References

[B1] Acosta-Jurado S. Alias-Villegas C. Navarro-Gómez P. Almozara A. Rodríguez-Carvajal M. A. Medina C. . (2020). Sinorhizobium fredii HH103 syrM inactivation affects the expression of a large number of genes, impairs nodulation with soybean, and extends the host-range to the Lotus japonicus. Environ. Microbiol. 22, 1104–1124. doi: 10.1111/1462-2920.14897 31845498

[B2] Beringer J. E. (1974). R factor transfers in Rhizobium leguminosarum. J. Gen. Microbiol. 84, 189–198. doi: 10.1099/00221287-84-1-188 4612098

[B3] Chang Y. L. Wang J. Y. Wang E. T. Liu H. C. Sui X. H. Chen W. X. (2011). Bradyrhizobium lablabi sp. nov. isolated from effective nodules of Lablab purpureus and Arachis hypogaea grown in Southern China. Int. J. Syst. Evol. Microbiol. 61, 2496–2502. doi: 10.1099/ijs.0.027110-0 21112989

[B4] Chen J. Y. Gu J. Wang E. T. Ma X. X. Kang S. T. Huang L. Z. . (2014). Wild peanut Arachis duranensis are nodulated by diverse and novel Bradyrhizobium species in acid soils. Syst. Appl. Microbiol. 37, 525–532. doi: 10.1016/j.ocsci.2024.09.004 24985193

[B5] Fabra A. Castro S. Taurian T. Angelini J. Ibañez F. Dardanelli M. . (2010). Interaction among Arachis hypogaea L. (peanut) and beneficial soil microorganisms: how much is it known? Crit. Rev. Microbiol. 36, 179–194. doi: 10.3109/10408410903584863 20214416

[B6] Figurski D. H. Helinski D. R. (1979). Replication of an origin-containing derivative of plasmid RK2 dependent on plasmid function provided in trans. Proc. Natl. Acad. Sci. U.S.A. 76, 1648–1652. doi: 10.1073/pnas.76.4.1648 377280 PMC383447

[B7] Hoffman B. M. Lukoyanov D. Yang Z. Y. Dean D. R. Seefeldt L. C. (2014). Mechanism of nitrogen fixation by nitrogenase: the next stage. Chem. Rev. 114, 4041–4062. doi: 10.1021/cr400641x 24467365 PMC4012840

[B8] Janczarek M. Rachwał K. (2013). Mutation in the pssA gene involved in exopolysaccharide synthesis leads to several physiological and symbiotic defects in Rhizobium leguminosarum bv. Trifolii. Int. J. Mol. Sci. 14, 23711–23735. doi: 10.3390/ijms141223711 24317432 PMC3876073

[B9] Kassaw T. Bridges W. J. Frugoli J. (2015). Multiple autoregulation of nodulation (AON) signals identified through split root analysis of Medicago truncatula sunn and rdn1 mutants. Plants 4, 209–224. doi: 10.3390/plants4020209 27135324 PMC4844323

[B10] Kilmartin J. R. Bernhardt P. V. Dhouib R. Hanson G. R. Riley M. J. Kappler U. . (2016). Effects of mutations in active site heme ligands on the spectroscopic and catalytic properties of SoxAX cytochromes. J. Inorg. Biochem. 162, 309–318. doi: 10.1016/j.jinorgbio.2016.04.015 27112898

[B11] Kosuta S. Hazledine S. Sun J. Miwa H. Morris R. J. Downie J. A. . (2008). Differential and chaotic calcium signatures in the symbiosis signaling pathway of legumes. Proc. Natl. Acad. Sci. U.S.A. 105, 9823–9828. doi: 10.1073/pnas.0803499105 18606999 PMC2474534

[B12] Li Y. H. (2019). Comparative genomic analysis of peanut bradyrhizobia reveals the genetic differences underlying two symbiotic phenotypes in peanut and mung bean and the evolution of Bradyrhizobium spp (Beijing: China Agricultural University). Ph. D Dissertation.

[B14] Li Y. H. Wang R. Sui X. H. Wang E. T. Zhang X. X. Tian C. F. . (2019). Bradyrhizobium nanningense sp. nov. Bradyrhizobium guangzhouense sp. nov. and Bradyrhizobium zhanjiangense sp. nov. isolated from effective nodules of peanut in southeast China. Syst. Appl. Microbiol. 42, 126002. doi: 10.1016/j.syapm.2019.126002 31362902

[B13] Li Y. H. Wang R. Zhang X. X. Young J. P. W. Wang E. T. Sui X. H. . (2015). Bradyrhizobium guangdongense sp. nov. and Bradyrhizobium guangxiense sp. nov. isolated from effective nodules of peanut. Int. J. Syst. Evol. Microbiol. 65, 4655–4661. doi: 10.1099/ijsem.0.000629 26409482

[B16] Liu H. Zhang C. Yang J. Yu N. Wang E. (2018a). Hormone modulation of legume-rhizobial symbiosis. JIPB 60, 632–648. doi: 10.1111/jipb.12653 29578639

[B15] Liu Y. H. Jiao Y. S. Liu L. X. Wang D. Tian C. F. Wang E. T. . (2018b). Nonspecific symbiosis between Sophora flavescens and different Rhizobia. Mol. Plant-Microbe Interact. 31, 224–232. doi: 10.1094/mpmi-05-17-0117-r 29173048

[B17] Mastronunzio J. E. Tisa L. S. Normand P. Benson D. R. (2008). Comparative secretome analysis suggests low plant cell wall degrading capacity in Frankia symbionts. BMC Genomics 9, 47. doi: 10.1186/1471-2164-9-47 18226217 PMC2266912

[B18] Nguyen H. P. Miwa H. Obirih-Opareh J. Suzaki T. Yasuda M. Okazaki S. (2020). Novel rhizobia exhibit superior nodulation and biological nitrogen fixation even under high nitrate concentrations. FEMS Microbiol. Ecol. 96, fiz184. doi: 10.1093/femsec/fiz184 31860058

[B19] Quandt J. Hynes M. F. (1993). Versatile suicide vectors which allow direct selection for gene replacement in Gram-negative bacteria. Gene 127, 15–21. doi: 10.1016/0378-1119(93)90611-6 8486283

[B20] Ren B. Wang X. Duan J. Ma J. (2019). Rhizobial tRNA-derived small RNAs are signal molecules regulating plant nodulation. Science 365, 919–922. doi: 10.1126/science.aav8907 31346137

[B21] Rostas K. Kondorosi E. Horvath B. Simoncsits A. Kondorosi A. (1986). Conservation of extended promoter regions of nodulation genes in Rhizobium. Proc. Natl. Acad. Sci. U.S.A. 83, 1757–1761. doi: 10.1073/pnas.83.6.1757 16593668 PMC323163

[B23] Safronova V. Sazanova A. Belimov A. Guro P. Kuznetsova I. Karlov D. . (2023). Synergy between rhizobial co-microsymbionts leads to an increase in the efficiency of plant-microbe interactions. Microorganisms 11, 1206. doi: 10.3390/microorganisms11051206 37317180 PMC10223793

[B22] Sambrook J. Fritsch E. F. Maniatis T. (1989). Molecular Cloning: A Laboratory Manual, 2nd Ed (Cold Spring Harbor, NY: Cold Spring Harbor Laboratory Press).

[B24] Schell M. A. (1993). Molecular biology of the LysR family of transcriptional regulators. Annu. Rev. Microbiol. 47, 597–626. doi: 10.1146/annurev.micro.47.1.597 8257110

[B25] Scheu A. K. Economou A. Hong G. F. Ghelani S. Johnston A. W. Downie J. A. (1992). Secretion of the Rhizobium leguminosarum nodulation protein NodO by haemolysin-type systems. Mol. Microbiol. 6, 231–238. doi: 10.1111/j.1365-2958.1992.tb02004.x 1545707

[B26] Shang J. Y. Zhang P. Jia Y. W. Lu Y. N. Wu Y. Ji S. . (2022). Coordinated regulation of symbiotic adaptation by NodD proteins and NolA in the type I peanut bradyrhizobial strain Bradyrhizobium zhanjiangense CCBAU51778. Microbiol. Res. 265, 127188. doi: 10.1016/j.micres.2022.127188 36152611

[B27] Shang J. Y. Zhang P. Jia Y. W. Lu Y. N. Wu Y. Ji S. . (2023). Scrutiny of NolA and NodD1 regulatory roles in symbiotic compatibility unveils new insights into Bradyrhizobium guangxiense CCBAU53363 interacting with peanut (Arachis hypogaea) and mung bean (Vigna radiata). Microbiol. Spectr. 11, e0209622. doi: 10.1128/spectrum.02096-22 36475917 PMC9927474

[B28] Shao L. Abdel-Motaal H. Chen J. Chen H. Xu T. Meng L. . (2018). Characterization of a functionally unknown Arginine-Aspartate-Aspartate family protein from Halobacillus andaensis and functional analysis of its conserved Arginine/Aspartate residues. Front. Microbiol. 9, 1–17. doi: 10.3389/fmicb.2018.00807 29922240 PMC5996927

[B29] Slonczewski J. L. Fujisawa M. Dopson M. Krulwich T. A. (2009). Cytoplasmic pH measurement and homeostasis in Bacteria and Archaea. Adv. Microb. Physiol. 55, 1–317. doi: 10.1016/s0065-2911(09)05501-5 19573695

[B30] Tate R. Riccio A. Iaccarino M. Patriarca E. J. (1997). A cysG mutant strain of Rhizobium etli pleiotropically defective in sulfate and nitrate assimilation. J. Bacteriol. 179, 7343–7350. doi: 10.1128/jb.179.23.7343-7350.1997 9393698 PMC179684

[B31] Tatsukami Y. Ueda M. (2016). Rhizobial gibberellin negatively regulates host nodule number. Sci. Rep. 6, 27998. doi: 10.1038/srep27998 27307029 PMC4910070

[B32] Tomlinson A. D. Hartung B. R. Day T. W. Merritt P. M. Fuqua C. (2010). Agrobacterium tumefaciens ExoR represses succinoglycan biosynthesis and is required for biofilm formation and motility. Microbiology 156, 2670–2681. doi: 10.1099/mic.0.039032-0 20576688 PMC3068688

[B33] Vincent J. M. (1970). A Manual for the Practical Study of Root Nodule Bacteria (Oxford: Blackwell).

[B34] Wang R. Chang Y. L. Zheng W. T. Zhang D. Zhang X. X. Sui X. H. . (2013). Bradyrhizobium arachidis sp. nov. isolated from effective nodules of Arachis hypogaea grown in China. Syst. Appl. Microbiol. 36, 101–105. doi: 10.1016/j.syapm.2012.10.009 23295123

[B35] Wu Y. Li Y. H. Shang J. Y. Wang E. T. Chen L. Huo B. . (2020). Multiple genes of symbiotic plasmid and chromosome in type II peanut Bradyrhizobium strains corresponding to the incompatible symbiosis with Vigna radiata. Front. Microbiol. 11, 1175. doi: 10.3389/fmicb.2020.01175 32655513 PMC7324677

[B36] Yan H. Xie J. B. Ji Z. J. Yuan N. Tian C. F. Ji S. K. . (2017). Evolutionarily conserved nodE, nodO, T1SS, and hydrogenase system in rhizobia of Astragalus membranaceus and Caragana intermedia. Front. Microbiol. 8, 2282. doi: 10.3389/fpls.2016.00309 29209294 PMC5702008

